# Transthyretin amyloid cardiomyopathy—new data regarding epidemiology, diagnosis, and treatment

**DOI:** 10.1093/eschf/xvaf002

**Published:** 2026-01-08

**Authors:** Michał Tkaczyszyn

**Affiliations:** Institute of Heart Diseases, Wroclaw Medical University, Wroclaw, Poland; Institute of Heart Diseases, Jan Mikulicz Radecki University Hospital in Wroclaw, Wroclaw, Poland

**Keywords:** Cardiomyopathy, heart failure, amyloidosis, transthyretin

The growing awareness of cardiologists regarding amyloid transthyretin cardiomyopathy (ATTR-CM), together with increasingly precise non-invasive diagnostic approaches—particularly the development of practical diagnostic algorithms that streamline and facilitate the interpretation of parallel nuclear medicine and haematology tests—and finally, the dynamic progress of targeted therapies demonstrated in large clinical trials, are collectively translating into improved outcomes for patients affected by this form of cardiomyopathy.^1^ ATTR-CM is no longer regarded as a ‘rare’ disease, as it was just one or two decades ago.^2,3^ Heart failure (HF)–related scientific journals remain a natural venue for disseminating new data on ATTR-CM; accordingly, the present overview summarizes recent publications addressing the epidemiology, diagnostics, and treatment—both specific and general HF-directed pharmacotherapy—of ATTR-CM, grouped within a dedicated Virtual Issue on this topic.

First, our understanding of ATTR-CM epidemiology continues to expand, with new data emerging from previously underrepresented regions such as the Middle East,^4^ which appear to confirm the trends reported by large collaborative consortia in Europe and the United States. It is also unsurprising that systematic evaluation of elderly patients presenting with left ventricular wall thickening and advanced heart rhythm disturbances—requiring permanent cardiac pacing—reveals many previously unrecognized cases of ATTR-CM.^5^ Beuthner *et al*.^6^ presented valuable insights into the predictors of histopathological confirmation of amyloidosis in endomyocardial biopsy samples obtained during transcatheter aortic valve implantation. Beyond a highly specific diagnostic algorithm incorporating echocardiographic, electrocardiographic, and neurohormonal parameters (albeit in a relatively low-prevalence population), the authors demonstrated that selected proteomic markers may also hold diagnostic value.^6^ This is not the only diagnostic algorithm recently published in *ESC Heart Failure*. Gioia *et al*.^7^ proposed a novel ‘AMY score’, derived from simple clinical and echocardiographic parameters, designed to identify wild-type ATTR-CM among patients with HF with preserved ejection fraction (HFpEF). Although ATTR-CM has long been primarily associated with the HFpEF phenotype—as illustrated by non-invasive screening studies in Japanese HFpEF cohorts^8^—growing evidence suggests that amyloidosis should also be considered as a potential aetiology in patients with the phenotype of mildly-reduced (HFmrEF) or reduced (HFrEF) ejection fraction.^9^

Advances have also been made in understanding left atrial pathology and its prognostic implications in ATTR-CM.^10^ Although one study included both ATTR-CM and light-chain cardiac amyloidosis (AL-CA) patients, left-sided ‘atriopathy’ appeared to differ between the two groups while still correlating with symptomatic HF across all subjects.^11^ In another study, Neculae *et al*.^12^ comprehensively compared ATTR-CM and AL-CA, defining potential cut-off values for cardiac and renal biomarkers as well as echocardiographic strain parameters, aiming for sensitive—though only moderately specific—differentiation of amyloid type before further targeted investigations. Interestingly, a recent study analysing electrocardiograms of cardiac amyloidosis patients^13^ showed that modern ECG-based criteria are not inferior to sophisticated echocardiographic analyses, highlighting the continued diagnostic value of this simple, widely available, and cost-effective modality. In terms of invasive diagnostics, Holt *et al*.^14^ reported findings from right heart catheterization procedure in 57 ATTR-CM patients, identifying pulmonary hypertension in 77% of cases—most frequently of isolated post-capillary or combined pre- and post-capillary type.

Numerous ongoing studies address pharmacotherapy in cardiac amyloidosis, both disease-specific and general HF–directed treatment stratified by ejection fraction. Kato *et al*.^15^ demonstrated high level of adherence to tafamidis therapy in a real-world Japanese ATTR-CM population, comparable to clinical trial data. Further, in one Swiss study, tafamidis therapy was shown to exert favourable myocardial anti-remodeling effects over 12 months, as documented by cardiac magnetic resonance imaging.^16^ These findings have important implications for maintaining or improving access to tafamidis, particularly for patients in the earlier or less symptomatic stages of disease. Efforts continue to define the optimal use of conventional HF medications, especially drugs, which show stronger evidence in HFrEF than in HFmrEF or HFpEF. The recently published Heart Failure Association of the European Society of Cardiology Clinical Consensus Statement summarized key recommendations on this topic.^17^ Recent *ESC Heart Failure* papers also provided new data on beta-blocker use in cardiac amyloidosis^18^ and on the effects of dapagliflozin in a murine model of ATTR-CM.^19^ Finally, while the prognostic value of cardiopulmonary exercise testing in cardiac amyloidosis has been further reviewed and summarized,^20^ novel data from Swedish and Danish investigators^21^ indicate that parameters reflecting myocardial energetic efficiency—derived from ¹¹C-acetate positron emission tomography—are also associated with outcomes in both ATTR-CM and AL-CA. These findings further support the concept of myocardial energetic impairment as an additional pathophysiological feature of cardiac amyloidosis. The potential translational implications of this phenomenon warrant further investigation.
